# The Role of Z Chromosome Localization Gene *psmd9* in Spermatogenesis of *Cynoglossus semilaevis*

**DOI:** 10.3390/ijms25126372

**Published:** 2024-06-09

**Authors:** Yuman Zhang, Yue Wang, Qian Liu, Hongyan Wang, Qian Wang, Changwei Shao

**Affiliations:** 1 State Key Laboratory of Mariculture Biobreeding and Sustainable Goods, Yellow Sea Fisheries Research Institute, Chinese Academy of Fishery Sciences, Qingdao 266071, China; ybb_zz@163.com (Y.Z.); lunawang16@163.com (Y.W.); liuqian97927@163.com (Q.L.); wanghongyan@ysfri.ac.cn (H.W.); 2 Laboratory for Marine Fisheries Science and Food Production Processes, Qingdao Marine Science and Technology Center, Qingdao 266237, China

**Keywords:** *psmd9*, spermatogenesis, sex chromosome, overexpression, RNAi

## Abstract

Proteasome 26S Subunit, Non-ATPase 9 (*psmd9*) plays an important role in the balance of protamine and the stability of the nucleolar structure during spermatogenesis. In this study, we cloned the *psmd9* of *Cynoglossus semilaevis* and analyzed its expression pattern. *psmd9* was identified on the Z chromosome of *C. semilaevis*, which is considered an interesting candidate gene for spermatogenesis. qRT-PCR and FISH experiments showed that the *psmd9* gene was significantly highly expressed in the testes. It is worth noting that the expression level of *psmd9* in male fish testes is significantly higher than that in pseudomales. In order to further explore the role of *psmd9* in spermatogenesis, a male testicular cell line was used as the experimental material. The results of the *psmd9*-RNAi and overexpression experiments showed that *psmd9* had a synergistic effect with spermatogenesis-related genes *dnd1*, *cfap69*, *dnah3* and *dnajb13*, but had an antagonistic effect with *ccne2*. Our findings offer a scientific foundation for comprehending the role of *psmd9* in the spermatogenesis regulatory network of *C. semilaevis*.

## 1. Introduction

Spermatogenesis is a dynamic, continuous and complex process, which involves key biological events, such as spermatogonial proliferation, the meiosis of spermatocytes, and sperm cell deformation and differentiation into mature sperm [1,2]. Similarly, during fish spermatogenesis, spermatogenic cells and somatic cells undergo highly specialized cell differentiation under precise molecular controls to produce functional sperm [3]. These processes of spermatogenesis are essential for continuous sperm production, which is dependent upon numerous factors, both intrinsic (Sertoli and germ cells) and extrinsic (androgens and retinoic acids) [4]. Studying the mechanism of spermatogenesis can not only deepen understanding of the reproductive biology of fish, but also provide a scientific basis for breaking through precision breeding technologies, such as sex control and artificial reproduction of fish [5,6], thus realizing efficient breeding and cultivating excellent varieties of sustainable aquaculture.

It is of great significance to analyze the regulation mechanism of cell fate in the process of spermatogenesis for realizing sperm production in vitro and accurate breeding with sex control. *Cynoglossus semilaevis* is an important economic fish in China, and there is a great sexual size dimorphism between males and females, with females being 2–4 times larger than males [7]. Genetic females may be transformed into phenotypic males, while the transformation from genetic male to phenotypic female is irreversible, which means that sex control breeding has great practical significance [7]. According to the statistical data of Li et al., in a natural state, the proportion of males is always higher than that of females [8]. The acquisition of a high female population is of great significance, so there is an urgent need to prepare superfemale individuals. Ideally, a pseudomale crossed with a normal female could produce a superfemale (WW), and then a superfemale crossed with a normal male (ZZ) could produce all-female offspring. Unfortunately, in the published literature, there are only rare records of the existence of W sperm in pseudomales [9,10]. Additional research into the molecular mechanism of spermatogenesis in *C. semilaevis* will offer valuable understanding of this intriguing biological process.

With the development of modern genetic science research, the acquisition of genome, transcriptome and protein sequencing may provide ideas for exploring the molecular mechanism of spermatogenesis. The construction of the genome of *C. semilaevis* provides help for the study of gene function [10]. Several genes located on the Z chromosome are thought to be related to the processes of spermatogenesis. For example, neuralized E3 ubiquitin protein ligase 3 (*neurl3*) plays a role in the spermatogenesis of *C. semilaevis* by regulating testis protein ubiquitination in a dosage-dependent manner [11]. In addition, the testis-specific protein kinase 1 gene (*tesk1*) is found mainly expressed in the sperm cells and mature sperm of *C. semilaevis*, which may play a role in the stages of sperm formation, and the spermatid perinuclear RNA-binding protein (*strbp*) is found mainly expressed at the stage of sperm cell metamorphosis, and may play a role in this stage [12,13]. Through the analysis of the gonad transcriptome and proteome of adult pseudomale fish and male fish, it was shown that the extracellular environment, such as growth factors and steroid hormones, are not the cause of sperm loss, and there may be many gene regulation defects during sperm maturation, which may lead to sperm maturation being blocked [14]. On the other hand, high-resolution single-cell RNA-seq maps from the testes of genotypic male and pseudomale fish revealed the dynamic changes in gene expression patterns during spermatogenesis in *C. semilaevis* [15]. In particular, the *psmd9* gene was significantly differentially expressed between pseudomales and males [15], and it was located on the Z chromosome [9], which indicates that *psmd9* might be related to spermatogenesis.

Proteasome 26S Subunit, Non-ATPase 9 (*psmd9*) is an active hub gene that links numerous pathways that are important for organelle structure, cell signaling, growth, survival and therapy resistance [16]. PSMD family members and their interacting molecules participate in the regulation of biological processes, such as the cell cycle, cell invasion and migration. In addition, knocking out *psmd9* inhibits cell proliferation, invasion and migration and induces G2/M cell cycle arrest [17]. It has been verified in an oligozoospermia model of mice that once the G2/M phase is successfully passed by adding follicle stimulating hormone (FSH) and estradiol (E2), haploid spermatogenic cells are increased and spermatogenesis is promoted [18]. However, the role of *psmd9* in the spermatogenesis of *C. semilaevis* remains unknown.

In this paper, the coding sequence (CDS) region of the *C. semilaevis psmd9* gene was cloned, and the expression level of the target gene in different tissues and different stages of the gonad was detected. In addition, overexpression and RNAi experiments were carried out on the testicular cell line of *C. semilaevis* to detect the relationship between *psmd9* and spermatogenesis-related genes. The results show that *psmd9* may affect the process of spermatogenesis by regulating the expression of multiple genes.

## 2. Results

### 2.1. Cloning and Sequence Analysis of psmd9

**Figure 1:**
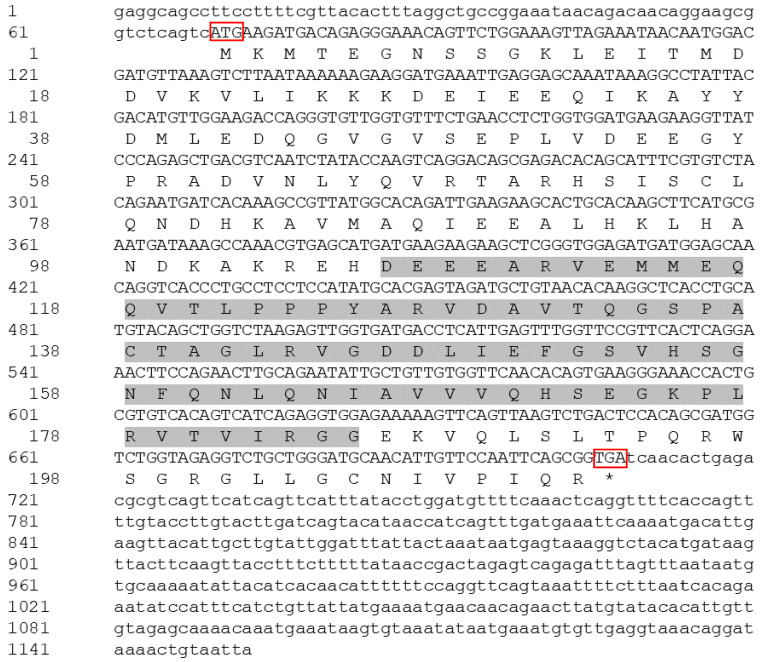
The cDNA sequence and deduced amino acid sequence of the *psmd9* gene. The red box represents the start codon and stop codon of *psmd9*, and the stop codon is indicated by an asterisk (*). The grey shadows represent the predicted protein domain.

The total length of the *psmd9* sequence is 1153 bp, including a 69 bp 5′UTR, 445 bp 3′UTR and 639 bp open reading frame. The sequence contains five introns and six exons encoding a total of 212 amino acids (Figure 1). The predicted protein has a relative molecular weight of 23.62 kDa and a theoretical isoelectric point (pI) of 5.41. The protein sequence analysis revealed that 106–185 aa have a PDZ domain.

### 2.2. Multisequence Alignment and Molecular Phylogenetic Analysis

**Figure 2:**
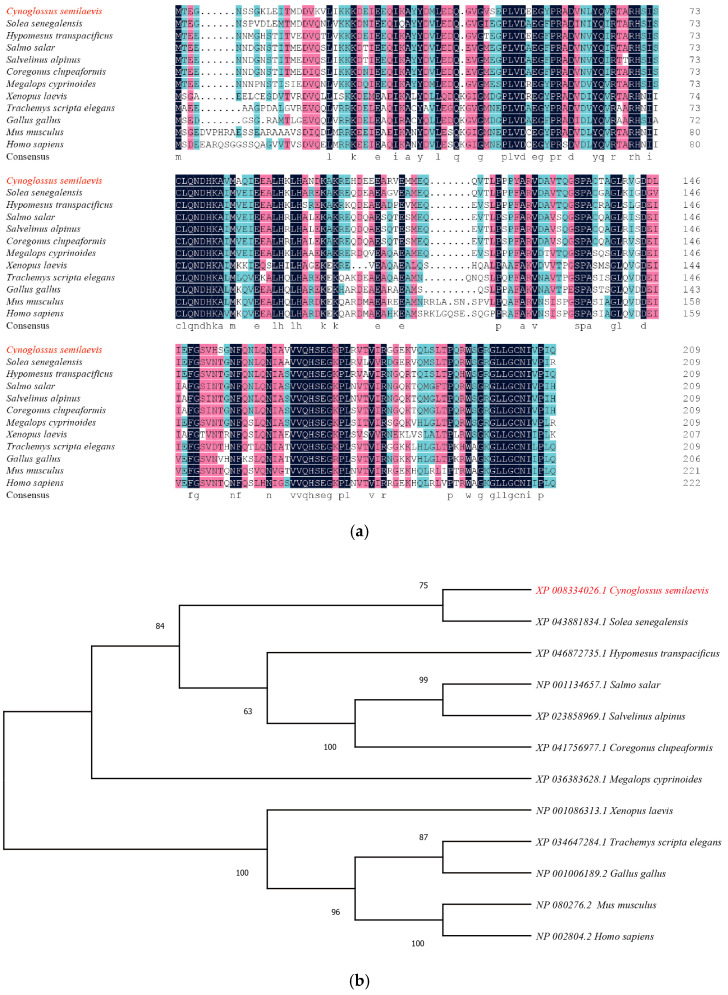
Multiple sequence alignments and phylogenetic tree of *psmd9* proteins. (**a**) DNAMAN was used to perform the multisequence alignment of the *psmd9* amino acid sequences. The blue boxes indicate 100% sequence identity between amino acid sequences, the pink boxes indicate more than 75% similarity between amino acid sequences, and the cyan boxes indicate 50% to 75% similarity between amino acid sequences. Note: *C. semilaevis* (Sequence ID: XP_008334026.1), *Solea senegalensis* (Sequence ID: XP_043881834.1), *Hypomesus transpacificus* (Sequence ID: XP_046872735.1), *Salmo salar* (Sequence ID: NP_001134657.1), *Salvelinus alpinus* (Sequence ID: XP_023858969.1), *Coregonus clupeaformis* (Sequence ID: XP_041756977.1), *Megalops cyprinoides* (Sequence ID: XP_036383628.1), *Xenopus laevis* (Sequence ID: NP_001086313.1), *Trachemys scripta elegans* (Sequence ID: XP_034647284.1), *Gallus gallus* (Sequence ID: NP_001006189.2), *Mus musculus* (Sequence ID: NP_080276.2) and *Homo sapiens* (Sequence ID: NP_002804.2). (**b**) Phylogenetic tree based on the *psmd9* amino acid sequence, using the neighbor-joining method. The number displayed on the branch node represents the guiding value (%). *C. semilaevis psmd9* is in red color.

To further understand the structure and function of *psmd9*, we compared the protein sequences of *psmd9* with those of other species. The results of multiple sequence alignments show that the amino acid sequence of *psmd9* has a 58.3~79.72% identity with that of other animals (Figure 2a). A phylogenetic tree was constructed based on the amino acid sequences, and the results show that *C. semilaevis* is clustered with *Solea senegalensis* (Figure 2b).

### 2.3. Expression Pattern of psmd9

**Figure 3:**
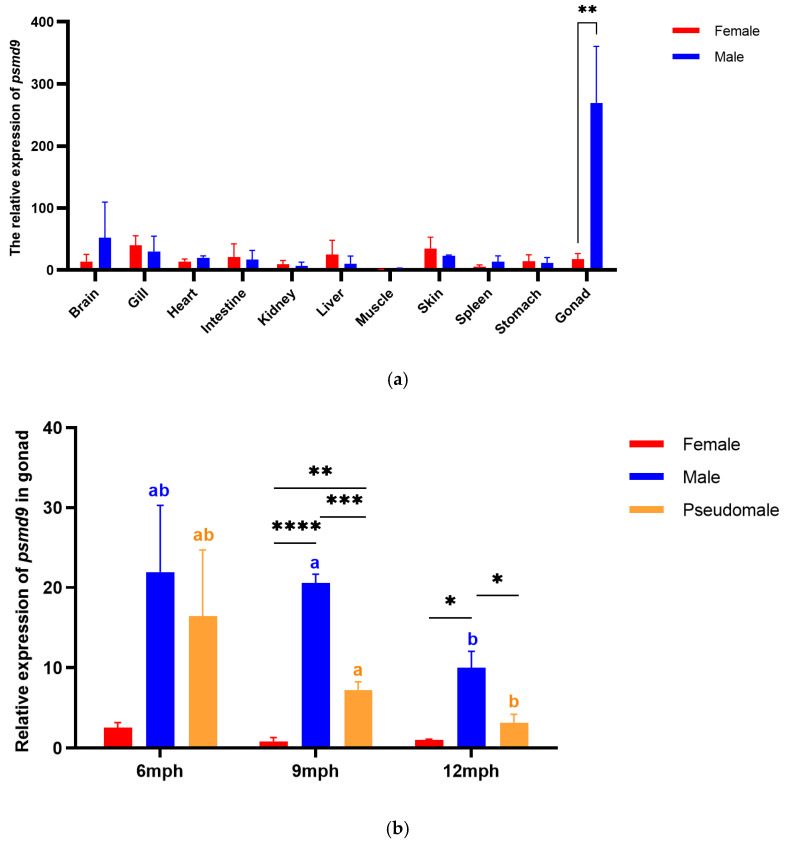
Expression of *psmd9* in *C. semilaevis*. (**a**) Expression of *psmd9* genes in adult *C. semilaevis* tissues. (**b**) Expression of *psmd9* from post-gonadal differentiation to maturation. Means ± SEM from three independent individuals (*n* = 3) are shown. *β-actin* was used as reference gene. Different letters indicate statistically significant differences (*p* < 0.05) in each sex among different stages. Asterisks indicate statistically significant differences (* *p* < 0.05; ** *p* < 0.01; *** *p* < 0.001; **** *p* < 0.0001) in each stage among different sexes.

In adult *C. semilaevis*, we measured the expression levels of *psmd9* in different tissues by qRT-PCR. The expression of *psmd9* was much higher in the testes than that in the ovaries. It also showed expression in the brain, gill, heart, intestine, kidney, liver, skin, spleen and stomach, and low expression only in the muscle (Figure 3a).

We further assessed *psmd9* expression patterns for different stages of the gonad. Overall, the expression level of *psmd9* in male testes was significantly higher than that in pseudomales, and both were higher than that in female ovaries at 9 mph (months post hatching) and 12 mph. The expression level of *psmd9* was highest at 6 mph, with no significant difference between the male and pseudomale testes. After that, *psmd9* expression decreased gradually from 6 mph to 9 mph to 12 mph, while the level of expression in males was always higher than that in pseudomales (Figure 3b).

### 2.4. FISH of psmd9 in Gonads

**Figure 4:**
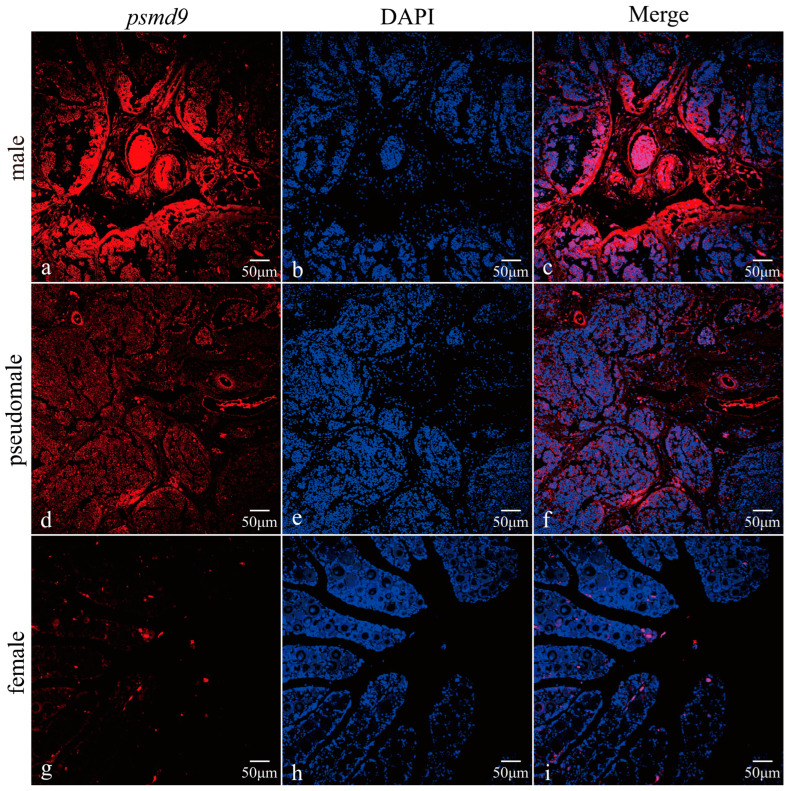
*psmd9* localization in the gonads of *C. semilaevis* at 9 mph. (**a**) Localization of *psmd9* detected using 5′Cy3-labeled fluorescent probe in male testes. (**b**) Spermatogenic cell nucleus and somatic cell nucleus were stained with DAPI in male testes. (**d**) Localization of *psmd9* detected using 5′Cy3-labeled fluorescent probe in pseudomale testes. (**e**) Spermatogenic cell nucleus and somatic cell nucleus were stained with DAPI in pseudomale testes. (**g**) Somatic nuclei were stained with DAPI in ovaries. (**h**) Localization of *psmd9* detected using 5′Cy3-labeled fluorescent probe in ovaries. (**c**,**f**,**i**) Combination of *psmd9* and nucleus localization. The blue signals represent nuclei and the red signals represent *psmd9* mRNAs, respectively.

We studied the location of *psmd9* in the gonads at 9 mph by fluorescence in situ hybridization (FISH). In 9 mph testes, mature sperm begins to be produced. *psmd9* has fluorescence signals in both the somatic cells and germ cells in the testes (Figure 4). Notably, the fluorescence signal is concentrated in the center of the testes of male fish (Figure 4c). Interestingly, the signal strength is significantly higher in males than in pseudomales. This is consistent with previous qRT-PCR results.

### 2.5. In Vitro Overexpression of psmd9 and its Effect on Other Genes

**Figure 5:**
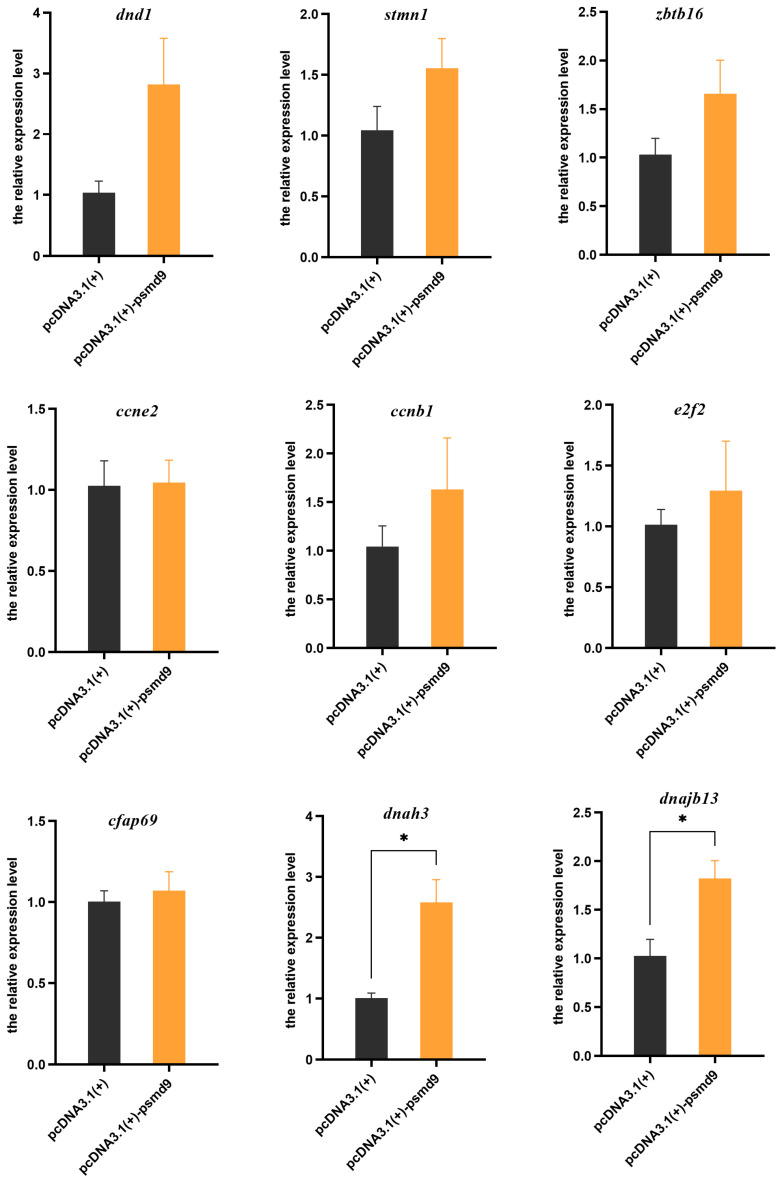
Relative expression level of germ cell marker genes in *C. semilaevis* testicular cell lines with overexpression of *psmd9*. Means ± SEM from three independent individuals (*n* = 3) are shown. *β-actin* was used as the reference gene. Asterisks indicate statistically significant differences (* *p* < 0.05).

To evaluate the role of *psmd9* in spermatogenesis, *psmd9* was overexpressed in the male testicular cell line of *C. semilaevis*, and its effect on other germ cell marker genes was detected. The results showed that compared with the control group, overexpressed *psmd9* significantly elevated the expression levels of spermatid/spermatozoa markers *dnajb13* and *dnah3* (*p* < 0.05). The expression levels of spermatogonia markers *dnd1*, *stmn1* and *zbtb16*; spermatocytes markers *ccne2*, *ccnb1* and *e2f2*; and spermatid/spermatozoa marker *cfap69* were not significantly affected (*p* > 0.05), even though there was an elevated trend (Figure 5).

### 2.6. psmd9 Knockdown and Its Effect on Other Genes

**Figure 6:**
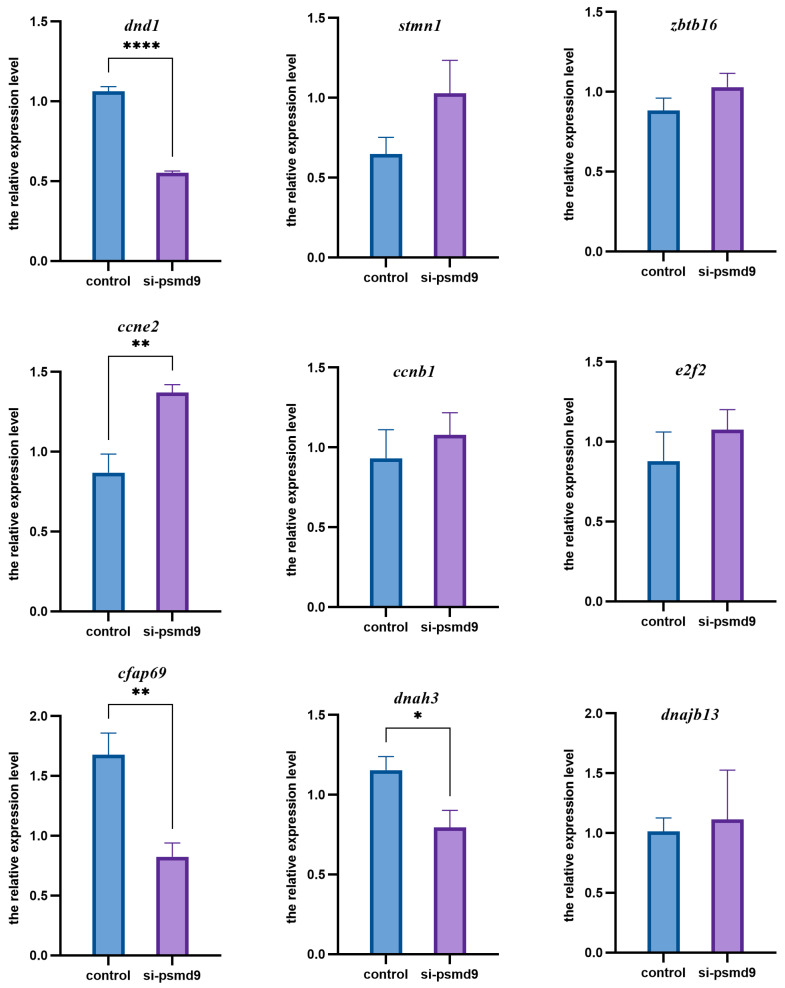
Relative expression level of germ cell marker genes in *C. semilaevis* testicular cell line with *psmd9* knockdown. Means ± SEM from three independent individuals (*n* = 3) are shown. *β-actin* was used as reference gene. Asterisks indicate statistically significant differences (* *p* < 0.05; ** *p* < 0.01; **** *p* < 0.0001).

We further performed a knockdown of *psmd9* by RNAi. The pre-experimental results showed that the efficiency of three mixed siRNAs had the best knockdown effect. In the following experiment, the transfection reagent group was set as the control group, and the transfection reagent plus siRNAs group was set as the experimental group. In the experimental group, spermatogonia marker *dnd1* and spermatid/spermatozoa markers *cfap69* and *dnah3* were significantly decreased (*p* < 0.05), while spermatocytes marker *ccne2* was significantly increased (*p* < 0.05). There was no significant change in spermatogonia markers *stmn1* and *zbtb16*, spermatocytes markers *ccnb1* and *e2f2*, or spermatid/spermatozoa marker *dnajb13* (Figure 6).

## 3. Discussion

In this study, the CDS sequence of the *C. semilaevis psmd9* gene is verified. *psmd9* encodes 212 amino acids, including five introns and six exons. It contains a PDZ conserved protein domain. The PDZ domain plays a role in transcription regulation, mRNA processing and editing, hormone and receptor activity and protein translation through its interaction with protein C-terminal residues [19]. A comparison of *psmd9* and similar proteins from different species shows a high degree of identity. In the phylogenetic tree, the predicted target gene proteins are clustered together in other teleost fishes. These results indicate that the PSMD9 protein is highly conserved in structure.

We further analyzed the expression pattern of the *psmd9* gene. The whole-tissue quantitative results showed that *psmd9* was highly expressed in the testis. It is worth considering that the significant difference between male and female gonads suggests that *psmd9* may play an important role in spermatogenesis. The results of the qRT-PCR and FISH on the *C. semilaevis* gonad showed that *psmd9* was highly expressed in the testis, especially in the male testis, and was significantly higher than that in the pseudomale testis. The fluorescence intensity showed that *psmd9* is expressed in both the somatic cells and germ cells of the testis, especially near the efferent duct. However, the expression of *psmd9* was extremely low in ZW pseudomales, which may be due to the fact that they have only one set of Z chromosomes.

In order to further understand the function of *psmd9*, we analyzed the expression of *psmd9* at different stages after gonad differentiation. The testis differentiation stage of *C. semilaevis* is slightly later than that of the ovary, and the efferent duct appears at 100 dph (days post hatching), and the formation of the seminal lobules at 150 dph marks the formation of the testis [20]. Because of the difference in water temperatures and seasons, there may be subtle differences in time [21]. At 6 mph, in the testis of *C. semilaevis*, there were more spermatocytes in the seminal lobule cavity, while spermatogonia were mainly at the edge of the seminal lobule [22]. At 9 mph, the seminal lobules in the testis are closely arranged, and the seminal vesicles in the seminal lobules contain spermatogonia, spermatocytes and sperm cells with synchronous development. At 12 mph, most of the sperm in the testis have matured, and the male fish enters the late stage of sperm release [23,24]. The qRT-PCR results after gonadal differentiation showed that *psmd9* was highly expressed at 6 mph and 9 mph, which indicates that *psmd9* may play an important role in spermatogenesis.

Sperm maturation involves the recombination of chromatin into highly dense nuclei, thus enhancing hydrodynamic ability and ensuring the integrity of the paternal genome [1]. This process is mediated by the replacement of histone by sperm nuclear basic protein (also known as protamine, PRM). Furthermore, the specific balance of protamine plays an important role in sperm DNA packaging [25]. In humans, small changes in the ratio of PRM1 to PRM2 can also lead to infertility [26]. The nucleolin *modulo* can mediate the transformation from histone to protamine during spermatogenesis in *Drosophila* [27]. PSMD9 is a companion to protease assembly. A cell knockout experiment confirmed that *psmd9* plays a significant role in maintaining protein balance [16]. In addition, PSMD9 interacts with several free ribosomes subunits and plays a role in the transport of ribosomal proteins to the nucleolus, which helps to maintain the structure and morphology of the nucleolar membrane-free compartment [28]. Therefore, *psmd9* may play a role in the balance of protamine during sperm maturation.

In order to further understand the role of *psmd9* in the spermatogenesis of *C. semilaevis*, overexpression and gene knockdown (RNAi) experiments were carried out on the testicular cell line of *C. semilaevis*, and the markers of germ cells at various development stages were detected. The types of germ cells were classified as follows: spermatogonia (*dnd1*, *stmn1* and *zbtb16*), spermatocytes (*ccne2*, *ccnb1* and *e2f2*) and spermatid/spermatozoa (*cfap69*, *dnah3* and *dnajb13*) [15]. In the experiment on the overexpression of *psmd9*, the expression level of spermatid/spermatozoa marker genes *dnah3* and *dnajb13* increased significantly. DNAH3 and DNAJB13, as dynein and flagella structural proteins, are considered to play an important role in sperm flagella movement [29,30]. DNAH3 is considered to be a dynein of flagella, and has ATPase activity. It produces a force towards the minus end of microtubules, depending on the release of ADP [29]. Thus, *dnah3* is related to sperm flagella assembly and participates in sperm motility [31]. The protein encoded by *dnajb13* plays an important role during the development of sperm flagella, through direct interaction with SEPTIN4 in the assembly and positioning of sperm annulus [30]. It is speculated that *psmd9* may play a synergistic role with *dnah3* and *dnajb13* in sperm flagella formation and sperm movement.

In the *psmd9*-RNAi experiment, the spermatid/spermatozoa marker gene *dnah3* was, correspondingly, significantly decreased. In addition, the spermatogonia marker gene *dnd1* and spermatid/spermatozoa marker gene *cfap69* decreased significantly, while the spermatocyte marker gene *ccne2* increased significantly. This may be due to the complexity of the spermatogenesis regulatory network, or the fact that other genes have similar functions as the target genes, which may have compensatory effects. *dnd1* encodes a protein that binds to the microRNA targeting sequence of mRNA, and positively regulates gene expression by inhibiting microRNA-mediated inhibition [32]. *dnd1* may play a role in the survival of primordial germ cells (PGC) [33]. The protein encoded by *ccne2* belongs to a highly conserved family of cyclin, which plays a role in the G1/S transformation of the cell cycle [34]. *cfap69* acts on the biogenesis of the sperm tail and the assembly of the central pair complex, which is located at the midpiece of the flagellum [35]. As a flagella-related protein, CFAP69 is essential for the assembly and stability of sperm flagella [36]. The knockdown of *psmd9* may promote spermatogonia to differentiate into spermatocytes. Therefore, *psmd9*, as a subunit of protease, cooperates with *dnd1*, *cfap69* and *dnah3* in the process of spermatogonia maintenance and sperm maturation, but antagonizes *ccne2* in the process of spermatogenesis.

It can be seen that *psmd9* mainly plays a role in PGC survival and sperm maturation. It has been reported that in mammals, *psmd9* silencing leads to the disintegration of the nucleolar structure, the accumulation of p53, and the slow growth of cells [28]. This phenomenon is similar to the pathophysiology of sperm damage after a vasectomy. A spermatogenesis disorder after a vasectomy is related to an increase in p53 and a decrease in proliferating cell nuclear antigen expression. Also, the P53-Bax pathway has been found to affect the apoptosis of some seminiferous tubules in the delayed period after a vasectomy [37]. Thus, the lack of *psmd9* may affect nucleolar instability, which may cause the accumulation of p53 protein and apoptosis in sperm. The specific mechanism of *psmd9* in apoptosis and spermatogenesis needs further study.

## 4. Materials and Methods

### 4.1. Fish and Tissue Collection

All the *C. semilaevis* used in this experiment were obtained from Huanghai Aquiculture, Ltd. (Yantai, China). After approval by the Institutional Animal Care and Use Committee (IACUC) of the Yellow Sea Fisheries Research Institute (CAFS) (Qingdao, China), the fish were collected and handled in accordance with the “Guidelines for Experimental Animals” of the Ministry of Science and Technology (Beijing, China). We dissected tissue samples from adult fish (12 mph), including the spleen, kidney, heart, liver, intestine, gill, muscle, skin, brain, and gonads, and immediately froze them with liquid nitrogen and stored them at −80 °C. The anatomical acquisition of tongue soles gonads at different stages of development was achieved, including at 6 months and 9 months and 12 months post hatching. After acquisition, one half of each sample was placed in liquid nitrogen and then stored at −80 °C for RNA extraction, and the other part was stored in 4% paraformaldehyde fixative for fluorescence in situ hybridization (FISH). The tailfins were cut and stored in ethanol for DNA extraction and genetic sex determination.

### 4.2. CDS Cloning of psmd9

The total RNA was extracted from the tissue samples according to the instructions for the TRIzol reagent (Invitrogen, Carlsbad, CA, USA). The RNA concentration and purity were determined by a Nano Drop 2000 spectrophotometer (Thermo, Waltham, MA, USA), and the integrity of the RNA was assessed with 1% agarose gel electrophoresis. The qualified RNA was stored at −80 °C. Then, the first-strand cDNA was synthesized using a HiScript III first-strand cDNA synthesis kit (+gDNA wiper) (Vazyme, Nanjing, China). A phenol–chloroform extraction method was used to extract the genomic DNA from the tailfin, and the genetic sex was identified by PCR using published methods and primers (sex F and sex R in Table 1) [38].

Based on the predicted sequences in the NCBI database (XM_008335804.2), we designed and synthesized primers (Table 1) to amplify *psmd9* from gonadal cDNA with PCR. After detection by 1% agarose gel electrophoresis, the product was purified using a FastPure^®^ Gel DNA Extraction Mini Kit (Vazyme, Nanjing, China). Then, the purified target fragment was ligated into a pEASY-T1 cloning vector (TransGene, Beijing, China), and the positive clones were selected for transformation into DH5α competent cells (TaKaRa, Shiga, Japan) for sequencing.

### 4.3. Real-Time Quantitative PCR

A Prime-Script RT Reagent Kit with gDNA Eraser (Takara, Dalian, China) was used to generate the cDNA templates from the total RNA. The primer sequences were designed using the target gene sequences as templates, and *β-actin* was used as an internal reference gene [39]. All the primers were synthesized by Ruibo, Ltd. (Beijing, China). According to the operation instructions for the QuantiNova SYBR Green PCR Kit (Qiagen, Hilden, Germany), qRT-PCR was performed with a LightCycler^®^ 480 PCR instrument (Roche, Basel, Switzerland). The reaction volume was 10 µL, including 5 µL 2 × SYBR Green PCR Master Mix, 0.4 µL primers (Table 1), and 1 µL of cDNA. The amplification conditions were as follows: 95 °C for 2 min, 40 cycles at 95 °C for 5 s, and 60 °C for 10 s. The melting curve reaction conditions were 95 °C for 15 s, 65 °C for 1 min, +0.11 °C/s to 95 °C, and 40 °C for 10 s. Three biological and three technical repetitions were tested. The 2^−∆∆Ct^ method was used for relative gene expression analysis [40].

### 4.4. Fluorescence In Situ Hybridization of psmd9

The testes at 9 mph were fixed with 4% PFA, embedded in paraffin and sliced into 3 µm sections. *psmd9*-specific fluorescent in situ hybridization probes labeled with 5′Cy3 were designed and synthesized. Fluorescence in situ hybridization was performed according to the instructions for the RNA FISH kit (Gene Pharma, Shanghai, China). The images were captured using a laser confocal microscope (Olympus, Tokyo, Japan) and analyzed.

### 4.5. Culture and Transfection of C. semilaevis Testicular Cell Line

The *psmd9* overexpression vector was constructed to verify the role of *psmd9*. The primers *psmd9*-EcoR1-F and *psmd9*-Xho1-R (Table 1) were used to amplify the target fragment for homologous recombination. The pcDNA3.1(+) vectors (Invitrogen, Carlsbad, CA, USA) were digested with EcoRI and XhoI. The pcDNA3.1(+)-*psmd9* plasmid was ligated using a ClonExpress Ultra One Step Cloning Kit (Vazyme, Nanjing, China). The recombinant plasmid was transformed into *Escherichia coli* and plated on a medium containing ampicillin. After 12 h, single colonies were selected for colony PCR, and the positive clones were sent for sequencing to verify whether the recombinant plasmid was correctly connected. The recombinant plasmid was extracted using an EndoFree Mini Plasmid Kit II (Tiangen Biotech, Beijing, China) for transfection.

The testicular cell line of *C. semilaevis* originated from the researchers’ laboratory and was cultured in L15 compound medium at 24 °C [41]. Taking an empty plasmid as the control group, Lipofectamine 3000 Reagent (Thermo Fisher, Invitrogen, Carlsbad, CA, USA) was used to transfect the recombinant plasmid into 6-well plates. Cells were collected 48 h after transfection, and the total RNA was extracted. The expression of the spermatogenesis-related genes was detected using qRT-PCR.

### 4.6. siRNA-Mediated Knockdown of psmd9 in Testicular Cell Line

Based on the sequence of *psmd9*, three specific siRNAs were designed and synthesized (Gene Pharma, Shanghai, China). The siRNA with the best knockdown effect was selected for further study. The testicular cell line of *C. semilaevis* was prepared according to the method mentioned above. In the control group, only the transfection reagents were treated without adding siRNAs. According to the previously established experimental protocol, the *psmd9* siRNA transfection and control transfection were performed in triplicate. After transfection for 48 h, the transfection efficiency was observed under a fluorescent inverted microscope and the cells were collected. The RNA was extracted according to the previous method and the expression of related genes was detected.

### 4.7. Statistical Analysis

All the experimental data were presented as means ± SEM, and all the experiments were performed in triplicate. Prism 6.0 (GraphPad Software, San Diego, CA, USA) was utilized for data processing, and *t*-tests and one-way ANOVA followed by Tukey multiple comparisons were conducted for the analysis. *p*-values less than 0.05 were considered statistically significant (* *p* < 0.05; ** *p* < 0.01; *** *p* < 0.001; **** *p* < 0.0001).

## Figures and Tables

**Table 1:** Primers used for cloning and gene expression analysis.

Primer Name	Sequence (5′–3′)	Purpose
*psmd9*-F	AACAGACAACAGGAAGCG	Partial fragment amplification
*psmd9*-R	CAGTTTTATCCTGTTTACCTC	
*psmd9*-EcoR1-F	tagtccagtgtggtggaattcATGAAGATGACAGAGGGAAACAGTT	Construction of overexpression vector
*psmd9*-Xho1-R	aacgggccctctagactcgagTCACCGCTGAATTGGAACAAT	
*psmd9*-qF	ACCAGGGTGTTGGTGTTTCTG	RT-qPCR
*psmd9*-qR	CGTTTGGCTTTATCATTCGCA	
*zbtb16*-qF	GCCGAAGTCAGTCAGATGGG	
*zbtb16*-qR	GCGTCCGATTTGTGATTGATA	
*dnd1*-qF	GCTGTGTAAGTTGCTTGATGTTGT	
*dnd1*-qR	GCCTGTGAAGGGTGCGG	
*stmn1*-qF	TTCCCTTGTCTGCCCCC	
*stmn1*-qR	CTTCTTTTTCATGTTCACGCTTC	
*ccne2*-qF	GAGCAGGAAAACAGTGGTGAAG	
*ccne2*-qR	GAATGTGTGGAAGTGACAGAAGG	
*ccnb1*-qF	ACCGCTTTCTTCAGGACCAC	
*ccnb1*-qR	TCTCCTCAAACTTAGACGCCAG	
*e2f2*-qF	CAGTGGCTGGTGGGAGATG	
*e2f2*-qR	CAGGGATTTCTCCGCTCG	
*cfap69*-qF	TGTTGAACCTGTTGGTGTTGATG	
*cfap69*-qR	GTGGTGGATTTGGGCGG	
*dnah3*-qF	TCTTTTGGGAGGTTAGGCAAG	
*dnah3*-qR	GTGGGATTTAGATGGGGTCG	
*dnajb13*-qF	TCTGGAGATGGCTCTGACTGG	
*dnajb13*-qR	GCACAATGTCGTTGATGGGTAT	
*β-actin*-qF	GAGTAGCCACGCTCTGTC	
*β-actin*-qR	GCTGTGCTGTCCCTGTA	
sex-F	CCTAAATGATGGATGTAGATTCTGTC	Sex identification
sex-R	GATCCAGAGAAAA-TAAACCCAGG	

## Data Availability

Data are contained within the article.
